# The effect of hyperbaric oxygen therapy in the inflammatory response in a mouse model of endometriosis: An experimental study

**DOI:** 10.18502/ijrm.v20i5.11049

**Published:** 2022-06-08

**Authors:** Dedy Syahrizal, Cut Mustika, Nanda Ayu Puspita, Mohammad Guritno Suryokusumo, Hendy Hendarto

**Affiliations:** ^1^Department of Biochemistry, Medical Faculty, Universitas Syiah Kuala, Banda Aceh, Indonesia.; ^2^Department of Public Health, Medical Faculty, Universitas Syiah Kuala, Banda Aceh, Indonesia.; ^3^Medical Hyperbaric Study Program, Faculty of Medicine, Universitas Airlangga, Surabaya, Indonesia.; ^4^Department of Obstetrics and Gynaecology, Faculty of Medicine, Universitas Airlangga, Surabaya, Indonesia.

**Keywords:** Endometriosis, Hyperbaric oxygenation, Inflammation, Hypoxia, Mouse.

## Abstract

**Background:**

Endometriosis pathogenesis is related to the inflammation shown by the secretion of pro-inflammatory mediators. This hypoxia condition can stimulate this condition.

**Objective:**

To investigate the effect of hyperbaric oxygen therapy (HBOT) on the inflammation reaction of endometriosis-induced mice.

**Materials and Methods:**

The animals were designated into 3 groups: I) the pre-test group, II) the post-test group receiving the HBOT, and III) the post-test group without HBOT. All groups were subjected to induction of endometriosis by xenotransplantation for 15 days. HBOT was given 30 min 3 times a day for 10 days. The evaluation of the HBOT effect was conducted by examining the endometrial tissue. The inflammation level was evaluated using the Klopfleisch semiquantitative scoring system (index remmele scale), whilst the expression of nuclear factor kappa (NF κ B) beta was measured by immunohistochemical staining.

**Results:**

The results showed that group I demonstrated the highest level of inflammation degree (9.41 ± 1.99) compared to the post-test groups (group II: 1.60 ± 0.53; group III: 2.42 ± 0.53). The HBOT-groups was found to have the lowest inflammation level compared to the non-HBOT group (p = 0.020). The results demonstrated that HBOT lowered the peritoneal inflammation degree caused by the endometrial lesion in mice. NF κ B expression on the post-test groups was significantly decreased, compared to the pre-test group (p ≤ 0.001), with a strong correlation between the NF κ B expression and the peritoneal inflammation level (p ≤ 0.001, r = 0.670).

**Conclusion:**

HBOT significantly reduced the inflammation level on the endometrial lesion in mice, involving the NF κ B pathway.

## 1. Introduction

Inflammation theory is one of the molecular mechanisms in the pathophysiology of endometriosis, which is demonstrated by the alteration of the physiological activities and infiltration of immune cells in the endometriosis sites (1, 2). Inflammation in endometriosis is also evident from the increase of secretion of pro-inflammatory mediators, such as cytokines and prostaglandin, in the site of lesion and peritoneal cavity (3). This condition can be stimulated by the lack, if not absence, of oxygen in the cell's environment, known as hypoxia condition.

Hypoxia stress potentiates the pro-inflammatory pathways in endometriosis by facilitating the accumulation of inflammatory molecules such as hypoxia-inducible factor-1 alpha (HIF-1α) and nuclear factor kappa beta, which lead to the downstream cascade effect of prostaglandin production (4-6). The deprivation of oxygen level also induces the imbalance of estrogen receptors resulting in the accumulation of HIF-1α in the endometriosis lesions (4, 5). Despite the inevitable role of hypoxia in endometriosis inflammatory response, only a few studies have been conducted to elucidate the molecular basis of hypoxia and identify the suitable treatment for the hypoxic condition in endometriosis.

Hyperbaric oxygen therapy (HBOT) is a medical tested to treat hypoxia-related medical conditions, including inflammatory diseases (6). A previous study demonstrated the positive effect of HBOT in reducing the pro-inflammatory cytokine HIF-1α in endometriosis lesions, possibly due to the clearance of HIF-1α molecules in an oxygen-rich environment (7). The effect of HBOT may lead to the alteration of another inflammatory cascade involving estrogenic receptors and the corresponding inflammatory mediator such as nuclear factor kappa (NF κ B), which decreases the inflammatory state in the lesions. NF κ B is an important factor in endometriosis pathophysiology, constitutively activated and highly expressed in endometrial lesions. This transcription factor serves a complex interaction with the steroid receptor, resulting in the maintenance of the inflammatory reaction in the lesion.

To show the role of HBOT in lowering the inflammatory state in endometriosis, we considered it necessary to evaluate the effect of HBOT in peritoneal inflammation and changes of the expression of NF κ B on the peritoneal tissue of mouse model endometriosis.

## 2. Materials and Methods

### Chemical products

All chemical substances used in this study were purchased from Sigma-Aldrich (St. Louise, MO, USA).

### Experimental animals

24 healthy adult female swiss albino mice (average age of 4 wk), with a weight range of 25-30 gr, were obtained from the Veteriner Farma Centre, Surabaya, Indonesia. Before starting the experiment, the animals were acclimatized for 1 wk for the adaptation process. The animal with more than 10% weight loss was excluded from the study.

### Experimental design 

Using a randomized controlled study design, the animals were designated into groups I, II, and III by simple random sampling. Each group consists of 8 animals. Group I was the pre-test group, group II was the post-test group receiving the HBOT, while group III was the post-test without HBOT. All animals were subjected to induction of apoptosis by xenotransplantation for 15 days. The pre-test group (I) and post-test group (II) were not given the HBOT treatment, while the group III was given HBOT treatment for 10 days. After the experiment, the group I was examined after endometriosis induction. The other 2 groups, II and III, were examined simultaneously after HBOT treatment for group III was completed.

### Preparation of endometrial cells

The endometrial cells were collected during the surgery from uterine adenomyosis women. The tissue was then washed using phosphate buffer saline (PBS) and homogenized using mortar and pestle. The ground tissue was washed in PBS and centrifuged at 2500 rpm twice to obtain the cell pellets. Subsequently, PBS containing 200 μg/mL of streptomycin and 200 IU/mL of penicillin were added into the cell pellets and incubated overnight.

### Induction of endometriosis 

The procedures for the xenotransplantation of endometrial cells on the mice were performed according to the previously published protocol (8). After the adaptation, each mouse was injected with 0.1 ml (10 mg/kg body weight) of cyclosporin A to suppress the animal's immune system. Then the mice were injected with 0.2 ml ethinyl estradiol at a dose of 0.2 μg/mouse by intramuscular injection. Subsequently, all mice were injected with the 0.1 ml endometrial cell suspension by intraperitoneal injection. On day 5, after the xenotransplantation, another dose of ethinyl estradiol was repeated. On day 15, the induction of endometriosis is completed.

### HBOT

One day after the endometriosis induction, the mice in group I was placed inside the hyperbaric chamber to receive the HBOT. The oxygen dose in the chamber was 100% O ${}_{2,}$ with a flow rate of 8-10 L/min. The hyperbaric oxygen treatment was given as 2.4 atm pressure for 3 × 30 min with a 5-min air break. The treatment was given for 10 days in a row.

### Histological evaluation 

After the experiment, each group was anesthetized and euthanized to collect the peritoneum tissue's histopathological samples from the peritoneum tissue. The areas on the peritoneum showing inflammatory signs (reddest part on the peritoneum) were sliced and preserved in 10% formalin before the staining process. Tissue slides were prepared by embedding the tissue in paraffin and sliced 4-6 µm thickness. Subsequently, the slides were stained in hematoxylin-eosin. Briefly, the tissue was deparaffinized in xylol for 5 min. This step was repeated twice. Then, the slides were immersed in a gradually reduced ethanol concentration for 1 min at each concentration; 100% ethanol twice, 95% ethanol twice, and 70% ethanol twice. Afterward, the slides were washed in water for 10 min before staining Mayer hematoxylin solution for 1 min. Then, the slides were counterstained in Eosin solution for 2 min and rehydrated in 95% alcohol and 100% ethanol for 3 min in each concentration of ethanol. Subsequently, xylol was added to the slides for 3 × 3 min, then air-dried and covered with cover glass. The examination of the slides was performed under a light microscope with 400 × magnification. The observation of the inflammation degree was using a modified Klopfleisch semiquantitative scoring system, which determines the inflammatory level according to the infiltration of inflammatory cells and the occurrence of granuloma mass (8, 9). As seen in table I, the scoring system summarizes the percentage of inflammatory cells (A) and the granuloma mass (B).

### Immunostaining for NF κ B

The tissue slides for immunohistochemistry staining were prepared following protocol described previously (10). The antibody and staining kits were purchased from Santa Cruz Biotechnology, CA, and the staining was performed according to the manufacturer's instructions. Briefly, the slides were fixed in acetone and blocked with 0.1% BSA. Peroxidase activities were depleted by using 0.3% hydrogen peroxide. Then, the slides were incubated in NF κ B antibody (ab16502) at room temperature for 3 hr. Following antibody staining, the slides were washed in PBS 3 times and incubated in the biotinylated secondary. The reaction visualization was done using a diaminobenzidine kit. The microscopic examination was performed to observe the expression of NF κ B expressed on the peritoneum tissue, and the score was measured semiquantitative according to the modified immunoreactive score (Index Remmele Scale) (11). The scoring system measures the percentage of positive cells (A) and the color intensity of the positive cells (Table II).

**Table 1:** The semi-quantitative scale of the inflammatory degree


**A - Inflammatory cell infiltration**	**B - Occurrence of granuloma mass**
**0 pts - not detected**	0 pts - not detected
**1 pts - less than 10 cells in 5 FOV (400 × )**	2 pts - detected
**2 pts - 11 to 50 cells in 5 FOV (400 × )**	4 pts - detected with abscess
**3 pts - 51 to 80 cells in 5 FOV (400 × )**	6 pts - detected with abscess and muscle tissue necrosis
**4 pts - more than 80 cells in 5 FOV (400 × )**	8 pts - detected with abscess and muscle tissue necrosis, and fibrosis
FOV: Field of view, pts: Points	

**Table 2:** Semiquantitative IRS scale of the expression of NF κ B


**A - Percentage of positive (+) cells**	**B - Intensity of the reaction color**
**0 pts - not detected**	0 pts - not detected
**1 pts - up to 10% cells with (+) reaction**	1 pts - low intensity
**2 pts - 11 to 50% cells with (+) reaction**	2 pts - moderate intensity
**3 pts - 51 to 80% cells with (+) reaction**	3 pts - intense
**4 pts - more than 80% cells with (+) reaction**	
IRS: Immuno reactive score, NF κ B: Nuclear factor kappa, Pts: Points	

### Ethical considerations

All experimental procedures followed the National guidelines for the experimental animal (Airlangga University, Indonesia) and were approved by the Animal Care and Use Committee of Airlangga University Surabaya, Indonesia (Code: ACUC no 497-KE).

### Statistical analysis

Data analysis was conducted by statistical analysis using a one-way ANOVA test. The correlation between the NF κ B level and the inflammation level was evaluated using Spearman's correlation test. Data were presented as a mean value with a standard deviation from 8 samples of each group. The statistical analysis used the statistical package for the social sciences (SPSS) software, version 19 (IBM Corp. Released 2010. IBM SPSS Statistics for Windows, Version 19.0. Armonk, NY: IBM Corp). A p-value < 0.05 was considered as significance.

## 3. Results

### Histopathology degree of peritoneal inflammation

**Figure 1:**
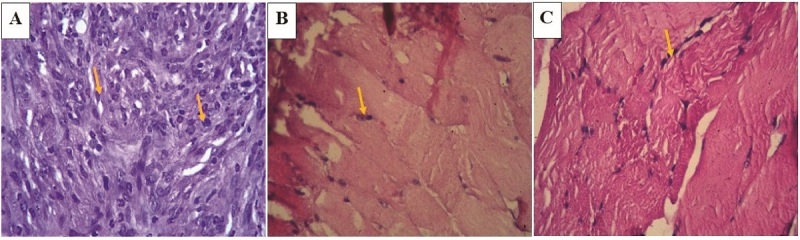
Histopathological degree of inflammation in group I (A), group II (B), and group III (C). The inflammation score in A is between 7 and 12, which is categorized as severe inflammation. Group II (B) and III (C) showed mild inflammation states, with inflammation scores between 1 and 3. The inflammation cells are shown by the arrows (400x magnification).

The implantation of endometrial cells has induced the inflammatory reaction in the mice peritoneal. The histopathological examination showed that the endometriosis lesions formed in all experimental animals within all groups, with various levels of inflammation degree (Figure 1).

**Figure 2:**
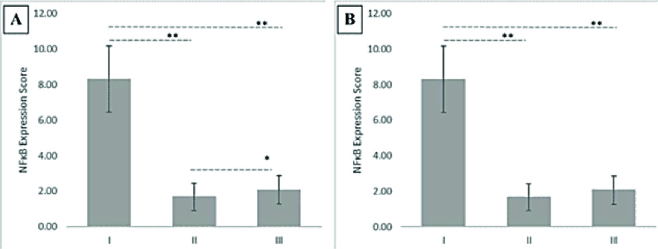
(A) The inflammation scores have shown the statistical difference of the inflammation degree between groups I, II, and III (*p = 0.500, **p = 0.020). Data are shown as the Mean ± standard error of the mean (n = 8). (B) The IRS scores have shown the statistical difference of the NF κ B expression between groups I, II, and III (*p = 0.500, **p = 0.001). Data are shown as the Mean ± standard error of the mean (n = 8). Groups were analysed using one-way Anova test, NF κ B*: *Nuclear factor kappa-light-chain-enhancer of activated B cells.

According to the Klopfleisch semiquantitative scores, the pre-test group (I) demonstrated the highest level of inflammation degree (9.41 ± 1.99) compared to the post-test groups (II: 1.60 ± 0.53; III: 2.42 ± 0.53). The group with HBOT (II) was found to have the lowest inflammation level compared to I (p = 0.020) and III (p = 0.020). The statistical difference between all groups is shown in figure 2. Overall, the results have demonstrated that HBOT has lowered the peritoneal inflammation degree caused by the endometrial lesion in mice.

### NF κ B expression

**Figure 3:**
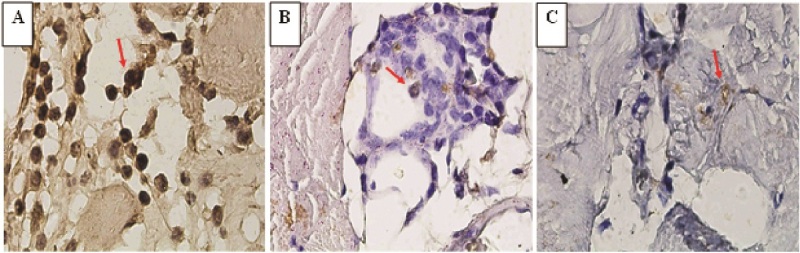
NF κ B expression on mice peritoneal tissue. Immunohistochemical positive immunoreactivity of NF κ B is shown by the arrows. In the pre-test group I (A), a large number of positive cells and high intensity of the reaction color showed a score between 7-12, indicating the increased expression of NF κ B. In the post-test groups II and III (B and C), only a small number of positive cells was found, accompanied by a low intensity of the reaction color.

The expression of NF κ B on the peritoneal tissue using immunohistochemical staining is shown in arrows (Figure 3). Based on the immuno reactive score (IRS) scoring method, the pre-test group (I) demonstrated the highest level of NF κ B expression (8.8 ± 2.32) compared to the post-test group (I and II). However, there is no significant difference between II and III (2.5 ± 2.66 and 2.7 ± 2.51, respectively).

### The correlation between NF κ B expression and the degree of inflammation

**Figure 4:**
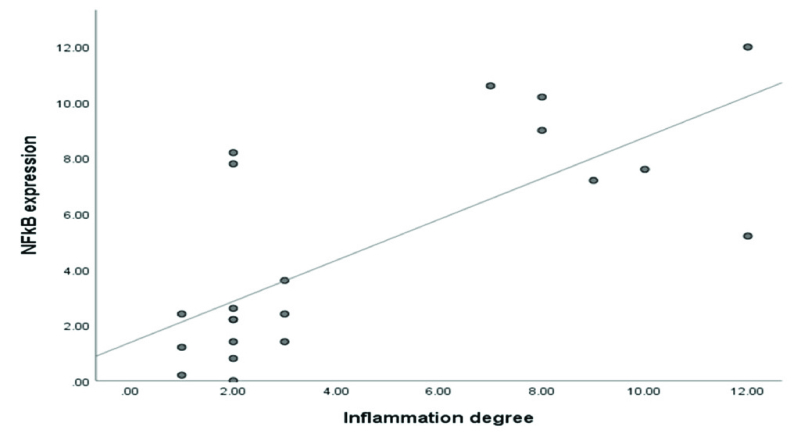
Correlation between NF κ B expression and inflammation degree in mice peritoneal tissue. The spearman's correlation test showed the correlation value r = 0.670 (p = 0.001).

The extent of NF κ B expression with the inflammation degree disclosed a pronounced positive correlation between the studied parameter (4). The correlation analysis resulted (Figure 4) in a coefficient value amounted to r = 0.670 (spearman's correlation).

## 4. Discussion

Our results demonstrated that a red and active lesion occurred as a sign of inflammatory reaction upon endometriosis induction in the mice peritoneum. Histopathological examination also demonstrated the high level of inflammation degree showed on the formed endometriosis lesions. The administration of HBOT for 2 wk on the endometriosis-induced mice significantly reduced the inflammation state of the formed lesion, compared to the group that did not receive HBOT (Klopfleisch scores 1.6 ± 0.53 and 2.4 ± 0.53, respectively). The results indicated a significant inflammation event occurred during the development of endometriosis lesions. The HBOT aimed at correcting the hypoxic environment within the cell environment increases the dissolved oxygen content in the circulation. It escalates the oxygen transfer to the inflamed cells (12, 13). It is well known that wound healing, particularly in inflammatory-related lesions, requires an adequate supply of oxygen tension because adequate oxygen supply is essential for the formation of collagen matrix and subsequent angiogenesis (10, 13). HBOT also reduces and eliminates the retention of CO ${}_{2}$ in the alveolar, which may help in increasing the oxygen content in the cells (14). In addition to this, HBOT has demonstrated a positive effect in reviving ischemic tissue by inhibiting the adhesion of neutrophil and post-ischemic vasoconstriction (15).

In the human endometrium, NF κ B protein can be expressed constitutively and play an inevitable role in the physiological changes on endometrial cells; the expression is exceptionally high during the secretion phase of the endometrial cycle (15). Activation of the NF κ B pathway is known as a response to several stimuli, including inflammatory stimuli Interleukin-1 (IL-1) and tumor necrosis factor alpha (TNF- α ), ROS, and hypoxia (16). In endometriosis pathogenesis, hypoxia occurs due to the disturbance of immune response, which leads to the aberrant secretion of inflammatory mediators from the endometrial tissue (17). On the other hand, the occurrence of hypoxia in a lesion will result in the exaggeration of the inflammatory response in the affected area, which may cause clinical manifestation such as chronic pain and other inflammatory-related symptoms in endometriosis (1, 18). Therefore, in the modality of endometriosis therapy, reducing the hypoxia condition may play a key role in inhibiting the progression of endometriosis lesion development by reducing the secretion and activation of inflammatory mediators and, more importantly, helping to ease the symptoms due to the inflammatory responses.

HBOT has been used in treating various medical conditions and has been proved to reduce the inflammatory mediator level (5, 19). Administration of HBOT for 2 hr a day in 6 wk on endometriosis-induced rats has demonstrated a significant lowering effect of the TNF- α level, leading to a remission of the endometriosis lesion due to the inhibition of NF κ B (19). The finding from the previous study also emphasized the positive effect of HBOT in reducing the NF κ B expression and other inflammatory cytokines in acute pancreatitis in rats. The same study showed that the level of nuclear factor of kappa light polypeptide gene enhancer in B-cells inhibitor, alphaIκBα, the co-activator of NF κ B, was notably reduced, suggesting that the mechanism of action of HBOT involving the NF κ B pathway (20). In our study, the administration of HBOT was given for 30 min 3 times a day for 10 days. The results demonstrated a significant decrease of NF κ B expression after the HBOT (IRS score 2.50 ± 2.66) compared to the expression before the treatment (IRS score 8.80 ± 2.320). However, the NF κ B expression showed no significant difference compared to the untreated group (IRS score 2.70 ± 2.51). The finding may show that in both groups receiving non-receiving HBOT, the low expression of NF κ B has given a clear sign of remission of the endometrial lesion. Our finding demonstrated that in the HBOT-receiving group, the inflammation level decreased significantly compared to the non-treated group (Klopfleisch scores 1.60 ± 0.53 and 2.40 ± 0.53, respectively). The immune systems may initiate the healing process without any treatment, by self-modulating the acute tissue repair process (21). Physiologically, this process will go through a complex cascade involving many stages of the healing-response phase. HBOT may speed up this process and increase the rate of healing response and the remission of an endometrial lesion in mice. In addition to this, our result has shown a strong correlation between NF κ B expression and inflammation degree, suggesting that NF κ B is an important factor in reducing the inflammation reaction. Therefore, our results have shown a positive effect of HBOT in reducing the inflammatory response in endometriosis. However, the level of NF κ B was yet to reach a significant confidence level compared to the untreated group, although previous studies have demonstrated constructive evidence on the HBOT effect in lowering the proinflammatory mediators.

In many studies on HBO treatment, the therapeutic effects were dose- and time-dependent (19, 20). Administration of HBO treatment on human blood-derived monocyte-macrophages for 3 hr reduced the Interleukin 1β synthesis, while prolonged HBOT for 12 hr increased the cytokine production (22). In indomethacin-induced enteropathy in rats, HBOT reduced the TNF- α expression after 12 hr and 24 hr treatment, but the effect was null after 48 hr. The same study demonstrated that HBOT showed no effect on the IL-1b production after 12 hr treatment. However, the positive effect was observed after 24 hr treatment, which showed that HBOT significantly lowered the IL-1β secretion (23). With regards to the HBOT effect on the inflammatory mediators in endometriosis, it has been reported that HBOT showed an observable effect on lowering the TNF- α levels after 6 wk of treatment (20). Therefore, all this evidence may explain the level of NF κ B shown from our result, which is likely related to the dose and duration of the HBOT used in this study.

## 5. Conclusion

Our study showed that HBO therapy reduced the inflammatory state in endometrial lesions, possibly via the alteration of the NF κ B pathway. Further investigation in the duration and dose of HBOT is needed to elucidate the molecular mechanism of HBOT in endometriosis. Our results have shown that HBOT significantly reduced the inflammation level on the endometrial lesions, with a low level of NF κ B expression.

##  Conflict of Interest

The authors declare that they have no competing interest.
